# Endoscopic/External Approaches in Otorhinolaryngology and Head and Neck Surgery

**DOI:** 10.1155/2015/958453

**Published:** 2015-02-23

**Authors:** Jan Betka, Karl Hörmann, Manuel Bernal-Sprekelsen, Jan Plzák

**Affiliations:** ^1^Department of Otorhinolaryngology and Head and Neck Surgery, 1st Faculty of Medicine, University Hospital Motol, V Uvalu 84, 150 06 Prague 5, Czech Republic; ^2^Department of Otorhinolaryngology and Head and Neck Surgery, University Hospital Mannheim, Theodor-Kutzer-Ufer 1-3, 68167 Mannheim, Germany; ^3^Department of Otorhinolaryngology and Head and Neck Surgery, University of Barcelona Medical School, Carrer Villarroel 170, 08036 Barcelona, Spain

Minimally invasive surgery has successfully entered the field of our subspecialty during the last decades. Endoscopic approach is nowadays well established in surgery of chronic rhinosinusitis (FESS, functional endoscopic sinus surgery) as well as in treatment of benign sinonasal diseases. Having gained experience with the endoscopic reconstruction of the anterior skull base [1] now an increasing amount of sinonasal malignancy is being treated endoscopically. Recently, the 4-hand expanded endoscopic approach through the nose to address tumors arising endocranially, with or without skull base involvement, has become extended, proving the nose to be a perfect access for tumors localized centrally and thus avoiding external approaches associated with higher morbidity [2].

Endoscopic laser microsurgery is widely used for benign and malignant laryngeal diseases. Initially, only early glottic or supraglottic tumors were chosen. However, more recently, also locally advanced tumors have been approached transorally with the CO_2_ laser [3, 4]. Also, tumors in the hypopharynx have been treated successfully, preserving the functional larynx and avoiding tracheotomies [5]. Compared to external approaches, transoral laser microsurgery has clearly shown reduced morbidity [6]; however, even in oncologically expert hands, a learning curve has to be expected [7].

Because of tendency to minimize invasiveness of surgery the endoscopy expands into other fields of otorhinolaryngology and head and neck surgery: neck soft tissue surgery (thyroid and parathyroid surgery), salivary gland surgery, skull base surgery, and so forth. But on the other side there are many examples when classical external approach is irreplaceable. There are even situations when both endoscopic and external approaches work effectively together.

The variety of indications and conditions that are now amenable to endoscopic approach underscores the substantial progress that has been made with endoscopic procedures in otorhinolaryngology, head and neck surgery. Just a few years ago, many of these cases still necessitated an external surgical approach.

Safe and effective endoscopy requires the use of a suitable endoscope (rigid, flexible, straight/angled, adjustable, equipped with camera/microscope, etc.) for adequate visualization of the operative field. An attached camera enables the assistant, nurse, and medical visitors to view the operative field and allows the procedure to be videotaped.

The inside diameter of the endoscope should conform to the size of the lumen in the patient. In the pediatric age group, the smaller caliber of the endoscope limits visualization of the surgical site and reduces the space available for instrument manipulations. Endoscopic procedures in infants are particularly difficult and require specialized training and experience [8].

Given the wide range of microinstruments now available, even extensive surgical procedures can be performed endoscopically. However, the surgeon should be prepared to change to an external approach at any time if it becomes necessary. In particular, when faced with complications such as bleeding, unfavorable anatomy, and loss of orientation, the surgeon should switch to an external approach without delay [9].

In this special issue, a dozen of papers are devoted to these characteristics. It contains review articles and original researches mainly on laryngology (laryngocele, hypopharyngeal diverticulum, subglottic stenosis, papillomatosis, and glottic carcinoma), rhinology + skull base (new technology using piezoelectric device for transnasal craniotomy and management of anterior skull base defect), and head and neck (oral + oropharyngeal carcinoma).

We hope that readers of Endoscopic/External Approaches in Otorhinolaryngology and Head and Neck Surgery will find in this special issue not only accurate data and updated reviews on the different surgical approaches in treatment of ENT disease, but also important questions to be resolved such as: how far may we sufficiently and safely reach to operate endoscopically and what are the real advantages and/or disadvantages of endoscopic versus external surgery?
